# Expectations and changing attitudes of bar workers before and after the implementation of smoke-free legislation in Scotland

**DOI:** 10.1186/1471-2458-7-206

**Published:** 2007-08-14

**Authors:** Shona Hilton, Sean Semple, Brian G Miller, Laura MacCalman, Mark Petticrew, Scott Dempsey, Audrey Naji, Jon G Ayres

**Affiliations:** 1 MRC Social and Public Health Sciences Unit, Glasgow, UK; 2 Department of Environmental & Occupational Medicine, University of Aberdeen, Aberdeen, UK; 3 Institute of Occupational Medicine, Edinburgh, UK

## Abstract

**Background:**

In Scotland on March 26, 2006 a comprehensive prohibition on smoking in all enclosed public places was introduced. This study examines bar workers' attitudes towards a smoke-free working environment.

**Methods:**

An intervention study comparing bar workers' opinions before and after the implementation of the smoke-free legislation. Bars were randomly selected in three Scottish cities (Glasgow, Edinburgh & Aberdeen) and towns (Aberdeenshire & Borders). Bar workers were recruited from 72 bars that agreed to participate from159 approached. Pre- and post-implementation attitudes towards legislation, second-hand smoke and smoke-free working environments were compared.

**Results:**

Initially the majority of bar workers agreed with the proposed legislation on smoking (69%) and the need for it to protect the health of workers (80%), although almost half (49%) thought the legislation would damage business. In 266 bar workers seen at both surveys, a significant positive attitudinal change towards the legislation was seen. Post-implementation, support for the legislation rose to 79%, bar workers continued to believe it was needed to protect health (81%) and concerns about the impact on business were expressed by fewer than 20%. Only the statement that the legislation would encourage smokers to quit showed reduced support, from 70% pre-implementation to fewer than 60% post-implementation. Initial acceptance was greater among younger bar workers; older workers, initially more sceptical, became less so with experience of the legislation's effects.

**Conclusion:**

This study shows that bar workers had generally positive attitudes towards the legislation prior to implementation, which became stronger after implementation. The affirmative attitudes of these key stakeholders are likely to contribute towards the creation of 'smoke-free' as the new social norm.

## Background

On March 26, 2006, Scotland followed Ireland, Norway and other countries in introducing a comprehensive prohibition on smoking in all enclosed public places [1]. The legislation was introduced primarily to reduce the harmful effects of second-hand smoke (SHS) exposure on the health of workers [2]. Recent estimates suggest that over 600 workers die annually in the UK as a result of their exposure to SHS at work, [3] with workers in the hospitality industry having some of the highest occupational SHS exposures [4]. Studies evaluating the health benefits of smoke-free legislation for workers exposed to SHS have shown positive health gains within short periods post-implementation [5]. However, a secondary benefit of the legislation is that people's attitudes towards the social acceptability of smoking may be influenced through efforts to 'de-normalise' smoking. In *A Breath of Fresh Air for Scotland *(2004), the Scottish Executive stated:

"Our long term aim is for no Scot to be exposed involuntarily to second-hand smoke at work or anywhere else and for them to choose to reject smoking as being an outdated and unfashionable practice which doesn't have a place within a healthy forward-looking nation." [6]

Successive UK governments have attempted to change attitudes to smoking and to reduce smoking prevalence through various tobacco control measures, including tax increases on tobacco, prohibiting tobacco advertising, provision of smoking cessation services and a succession of hard-hitting anti-smoking campaigns. Although there is good evidence that these measures increase the social unacceptability of smoking [7] and reduce smoking prevalence, [8] smoking remains an expensive habit imposing a huge economic burden on the NHS [9] and society through ill health, premature death and increased inequalities in health [10].

Experience from other countries suggests that smoke-free laws are associated with reduced adolescent smoking, [11,12] reduced tobacco sales, [13] increased smoking cessation [14] and changes in attitudes towards the acceptability of smoke-free workplaces. A study of California bar owners and staff surveyed before and after smoking restrictions were implemented found that the proportion preferring to work in a smoke-free environment increased from 17% before the restrictions to 51% afterwards [15].

Smokers' attitudes have also changed. In Ireland, a telephone survey of adult smokers (n = 769) found that support for smoking legislation in workplaces increased from 43% pre-implementation to 67% post-implementation [16] and, in New Zealand, bar managers who approved of smokefree bars increased from 44% to 60% and public support rose from 56% to 69% [17]. A population-based telephone study conducted in Canada investigated differences between smokers' and non-smokers' attitudes and behaviours to smoking and smoking restrictions [18]. They found that non-smokers whom they described as most 'adamant' about the benefits of legislation and aware of the harmful effects of SHS were most likely to refuse to sit in a smoking section of a restaurant. These individuals were also likely to be younger, to be better educated, and to be less likely to live with a spouse. In contrast, smokers who were classified as 'adamant smokers' were less likely to acknowledge the health risks of smoking to themselves and others, less supportive of the smoking legislation, and were older.

In the UK little is known about the views of workers in the hospitality sector in relation to SHS and legislation to prohibit smoking in enclosed public places. A recent postal survey of 1568 London casino workers found that 91% of workers wanted to change jobs because of SHS, [19] and a telephone survey of 545 Scottish bar workers indicated that 92% of bar workers thought smoke-free legislation would have positive effects on their health[20] The current study is part of the Bar Workers' Health and Environmental Tobacco Smoke Exposure (BHETSE) project, which forms one aspect of a comprehensive evaluation programme of the smoke-free legislation in Scotland [21]. This paper offers the first Scottish perspective on changes in bar workers' attitudes towards the legislation before and after its implementation, the health effects of SHS and working in a smoke-free environment.

## Methods

All bars from within designated postcode (ZIP) areas within three large cities (Glasgow, Edinburgh, Aberdeen) and small towns (population <3000) in the Aberdeenshire and Borders areas within Scotland were entered in to a study database. From a total of 861 available bars covering a broad range of socio-economic areas and types of bars in urban, semi-urban and rural settings, a total of 159 bars were selected at random in sequence in order to recruit a quote of 120 participants in each of the three areas. Each selected bar was contacted by telephone and invited to take part in the study. Bar managers who expressed interest were sent letters and other material describing the study to distribute to all their bar staff. If permission was granted by bar managers, bar visits were conducted by the researcher working in each city at prearranged times to maximise the number of bar staff recruited at each visit. From the 159 bars we contacted, 72 (45%) bars agreed to participate. Participation rates were highest in Aberdeen/shire with 23 bars from 45 contacted (51%), with Edinburgh/Borders (34/73; 47%) and Glasgow (15/41; 37%) having lower participation rates. The primary reasons given for non-participation from over 90% of bar managers was lack of time or being too busy to participate.

We carried out convenience sampling between January 7th and March 25^th ^2006 of a total of 371 bar workers (including managers, owners and bar staff), who were available and willing to take part at the time of our visits, across a range of weekday and weekend shift times. The bar workers completed a baseline survey in the three months leading up to the legislation (26^th ^March 2006) and were followed up between May and July 2006 to assess any change in attitudes towards the legislation, and to working in a smoke-free environment. Follow-up interviews were carried out by contacting the original bar where the worker was seen and arranging a suitable visit time, or by contacting the worker at their home address/telephone number if they no longer worked at the original bar.

During the visits bar staff completed a health and attitudes questionnaire, carried out lung function testing and provided a saliva sample for cotinine analysis. Lung function and salivary cotinine data will be reported separately. The questionnaire asked bar workers to rate on a five-point ordered scale their views on smoking and on the forthcoming smoking restrictions (Tables 2). These same questions (modified only by the tense of the question) were asked again at the follow up visit (Table 3). The survey items were adapted from questions used in the All Ireland Study of Bar Workers' Respiratory Health and will enable direct comparison with that dataset [5].

**Table 2:** Distribution of attitudes in the initial survey, and comparison of pre- and post-legislation attitudes in those followed up: for each question, percentages giving each response. N is the number of people analysed.

	**Question**	**N**	**Response (%)**				
			**1**	**2**	**3**	**4**	**5**
A	The ban on smoking will have a negative effect on business for public bars	370	16.2	33.0	33.2	14.9	2.7
B	The smoking ban is an unfair restriction on smokers	368	12.8	16.3	16.6	34.5	19.8
C	Fewer people will visit public bars after the ban on smoking	368	11.1	30.2	28.0	24.7	6.0
D	The smoking ban will make smokers smoke more at home	370	14.6	32.7	28.9	21.1	2.7
E	The smoking ban will result in jobs being lost	368	8.4	18.2	32.9	36.4	4.1

			Response* (%)				
			**5**	**4**	**3**	**2**	**1**

F	Smoke free public bars will make visits to them more comfortable	370	1.6	8.4	14.3	32.7	43.0
G	The smoking ban will encourage smokers to quit	370	0.8	9.2	20.8	50.5	18.6
H	The smoking ban is needed to protect the health of workers	370	0.5	7.3	11.9	41.4	38.9
I	Do you agree with the proposed ban on smoking in public bars?	368	9.8	12.0	9.5	27.2	41.6

* Response: 1-Strongly Agree, 2-Agree, 3-Undecided, 4-Disagree, 5-Strongly Disagree

**Table 3:** Comparison of pre- and post-legislation attitudes in those followed up: for each question, percentages giving each response. N is the number of people analysed.

	**Question**	**N**	**Period**	Response* (%)					**Mean Shift**	U-1** p-value
				**1**	**2**	**3**	**4**	**5**		
A	The ban on smoking will have (has had) a negative effect on business for public bars	265	Pre	15.8	34.7	32.8	15.5	1.1	1.00	<0.001
			Post	4.5	14.7	22.2	41.9	16.6		
B	The smoking ban is an unfair restriction on smokers	263	Pre	12.2	16.3	16.3	35.0	20.2	0.24	0.018
			Post	6.5	14.4	17.1	38.4	23.6		
C	Fewer people will (now) visit public bars after (because of) the ban on smoking	264	Pre	10.6	31.4	27.7	25.8	4.5	0.78	<0.001
			Post	4.9	9.8	21.6	47.0	16.7		
D	The smoking ban will make (has made) smokers smoke more at home	265	Pre	14.0	32.8	31.3	19.6	2.3	0.32	<0.001
			Post	7.5	18.0	48.3	23.8	2.3		
E	The smoking ban will result (has resulted) in jobs being lost	265	Pre	8.3	17.7	34.7	35.1	4.2	0.55	<0.001
			Post	2.6	4.2	32.8	47.5	12.8		

				Response* (%)						
				**5**	**4**	**3**	**2**	**1**		

F	Smoke free public bars will make (have made) visits to them more comfortable	265	Pre	1.5	9.4	15.1	30.2	43.8	0.20	0.018
			Post	1.1	6.0	11.3	29.8	51.7		
G	The smoking ban will encourage (has encouraged) smokers to quit	265	Pre	0.8	7.9	21.9	51.3	18.1	-0.28	<0.001
			Post	3.0	11.7	26.4	49.8	9.1		
H	The smoking ban is (was) needed to protect the health of workers	265	Pre	0.4	9.1	12.8	39.6	38.1	0.03	0.972
			Post	1.9	6.4	10.6	42.6	38.5		
I	Do you agree with the proposed ban on smoking in public bars?	263	Pre	9.9	12.5	9.9	27.0	40.7	0.32	0.017
			Post	5.3	6.5	9.9	31.9	46.4		

* Response: 1-Strongly Agree, 2-Agree, 3-Undecided, 4-Disagree, 5-Strongly Disagree

** Wilcoxon-Mann-Whitney U-test of equal medians. Tests the overall change in attitude for each question.

The study protocol was examined by the Grampian University Hospital Trust Ethics Committee and ethical overview was provided by the study Advisory Committee Group.

### Data analysis

Pre- and post-implementation responses on the five-point scale were tabulated, sub-divided by age group (up to 30 years and over 30 years) and by smoking status. Since occasional and ex-smokers are likely to be heterogeneous groups, and since their numbers were small, results by smoking status are presented only for smokers and non-smokers. (In general, attitudes for the omitted groups lay between those of smokers and non-smokers).

Change on the five-point scale between the responses given before and after the legislation was calculated for each question and for each respondent. Change in opinions after the legislation were summarised by averaging these numbers to produce a 'mean shift' parameter; in each case, the sign was arranged to be positive for a change in attitude more favourable to anti-smoking legislation. The statistical significance of these shifts, and of comparisons between the distributions of changes by age, gender and by smoking, was assessed using the Wilcoxon-Mann-Whitney U-test, [22] a non-parametric test for the difference in the median of two distributions.

## Results

### Bar worker characteristics

Of the 371 bar workers who participated in the baseline survey, 266 (72%) were seen at both baseline and follow-up. Table 1 compares subjects followed up with those lost to follow up. The two groups did not differ significantly in gender, smoking status, level of education or attitudes at baseline. However, there were differences in follow-up rate by age and location. The proportion of those aged over 30 who were successfully followed up was 81% for all three locations. The follow-up rate among those aged 30 or younger was 74% for Aberdeen/shire and Glasgow but only 59% for Edinburgh/Borders, reflecting Edinburgh's younger and more transient bar worker population. The broad similarities in demographics between those followed up and those lost to follow up suggest that the continuing participants may be considered as broadly representative.

**Table 1:** Characteristics of bar workers surveyed at follow-up and lost to follow-up.

**Characteristics**	**Surveyed at Follow-up**		**Lost to Follow-up**	
	**Mean**	**(Range)**	**Mean**	**(Range)**
**Age (at initial survey)**	28.2	(14, 66)	25.3	(18, 71)
**Years worked in bars (at initial survey)**	7.5	(0.15, 43)	7.0	(0, 43)

	**Number**	**(%)**	**Number**	**(%)**
**Sex**				
Male	130	(49)	61	(58)
Female	136	(51)	44	(42)
**Smoking Status**				
Regular Smoker	108	(41)	51	(49)
Occasional Smoker	32	(12)	11	(10)
Ex-Smoker	47	(18)	11	(10)
Non-Smoker	77	(29)	32	(30)
*Not answered*	*2*	*(1)*	*0*	*(0)*
**Location**				
Aberdeen/shire	91	(34)	29	(28)
Glasgow	91	(34)	30	(29)
Edinburgh/Borders	84	(32)	46	(44)
**Education Level**				
School	63	(24)	18	(17)
FE College	73	(27)	34	(32)
University	120	(45)	50	(48)
Postgraduate	10	(4)	3	(3)
				
**Total**	**266**		**105**	

### Bar workers' attitudes

Table 2 summarises the attitudinal responses of the 371 bar workers initially surveyed, while Table 3 compares the pre- and post-implementation responses for the 266 successfully followed up. The direction of responses has been organised so that the attitudes towards the legislation become more positive as one reads from left to right in the table.

#### Pre-legislation attitudes

Bar workers' attitudes at baseline were generally favourable towards the impending legislation. Seventy-six percent expressed agreement that smoke-free bars would be more comfortable places to visit as opposed to 10% who disagreed (question F). Similarly, high percentages of bar workers agreed that the ban would encourage smokers to quit (69%) and that there was a need for the ban to protect workers' health (80%) with only small proportions (10% and 8% respectively) rejecting these views (questions G, H). The baseline survey does show evidence of bar workers concerns, primarily over economic issues relating to customer numbers and jobs. Forty-nine percent of bar workers thought the ban would have a negative effect on business, 41% agreeing that the ban would reduce customer numbers (question A). However, only 27% of bar workers believed it would lead to job losses with 40% believing this was unlikely (question E). Overall, more than two thirds (69%) of bar workers expressed either agreement (27%) or strong agreement (42%) with the proposed legislation (question I).

#### Changes in attitudes

A positive and significant attitudinal change towards the smoking legislation among bar workers is seen in table 3. This was most noticeable in relation to their pre-implementation concerns about the legislation being bad for business. Almost half of respondents thought the legislation would damage business pre-implementation, but this dropped to fewer than 20% post-implementation (question A). Among the remaining respondents there was optimism that jobs were secure: this was reflected in a rise from 40% to 61% of respondents disagreeing with the statement that the smoking legislation would result in jobs being lost (question E). While it is possible that some of the 28% lost to follow-up may have lost their jobs as a consequence of the legislation, it is likely those remaining would have been aware of job losses and responded accordingly. Before the legislation nearly half (47%) of the bar workers thought that the legislation would displace smoking to the home, falling to 1 in 4 (24%) post legislation (question D). Before the legislation's implementation 40% of workers feared that fewer people would visit public bars after its introduction, but at follow-up only 14% agreed with this statement (question C). Pre-implementation nearly a third of bar workers thought the legislation was unfair on smokers, but this reduced to 21% at follow-up (question B). There was a negative shift in attitudes to the likely effect of the smoking legislation on encouraging smokers to quit (question G), which may have reflected their observations that their colleagues and patrons continued to smoke: an initially high degree of optimism of nearly 70% of respondents reduced to fewer than 60% post-implementation. There was also a change in attitude in relation to general support for the introduction of the legislation: the majority (69%) agreed initially with the proposed legislation. (question I) At follow-up the percentage agreeing with the legislation rose further to 79%, and the percentage disagreeing reduced to 12%. There was almost no change in views on the statement that the smoking legislation was needed to protect the health of workers from a very high 4 in 5 agreeing both pre- (80%) and post-legislation (81%) (question H). There was also a strengthening in agreement that smoke-free legislation would make visits to bars more comfortable, increasing from75% before to 81% after the legislation (question A).

### Age and attitudes towards legislation

Table 4 shows the attitudes towards each question, pre- and post-implementation, split by age group up to and above 30 years. Not all respondents answered every question. For questions A, C, D, E, there was a significant positive (i.e. pro-legislation) shift in attitudes in both age groups. For questions B, F, G, I, a significant shift was seen only in the older group. There was no significant shift for question H, but both groups had anyway been initially supportive of the need to protect workers' health, so there was less opportunity here for increased support. Whether or not significant, the shifts for all of these eight questions were in the positive direction, except for question G, asking whether the smoking legislation will encourage (has encouraged) smokers to quit smoking. Here the shift was negative, although significant only for those 30 and under [see Additional file 1].

**Table 4:** Attitudes pre- and post-legislation, split by age group: for each question, percentages giving each response. N is the number of people in each age group.

	**Question**	**Age Group**	**N**	**Period**	Response*					**Mean Shift**	U-1** p-value	U-2*** p-value
					**1**	**2**	**3**	**4**	**5**			
A	The ban on smoking will have (has had) a negative effect on business for public bars	≤30	192	Pre	11	31	39	17	2	0.90	<0.001	0.01
				Post	4	14	22	43	17			
		>30	73	Pre	27	44	18	11	0	1.27	<0.001	
				Post	5	18	23	38	15			
B	The smoking ban is an unfair restriction on smokers	≤30	190	Pre	6	13	17	41	24	0.14	0.277	0.018
				Post	4	11	18	40	27			
		>30	73	Pre	29	25	15	21	11	0.49	0.026	
				Post	14	25	14	34	14			
C	Fewer people will (now) visit public bars after (because of) the ban on smoking	≤30	192	Pre	6	31	28	30	6	0.63	<0.001	0.002
				Post	5	9	24	44	18			
		>30	72	Pre	22	33	28	15	1	1.19	<0.001	
				Post	6	11	15	54	14			
D	The smoking ban will make (has made) smokers smoke more at home	≤30	192	Pre	13	30	33	22	2	0.27	0.007	0.186
				Post	8	18	46	25	3			
		>30	73	Pre	16	41	26	14	3	0.45	0.001	
				Post	7	18	55	19	1			
E	The smoking ban will result (has resulted) in jobs being lost	≤30	192	Pre	4	15	33	42	6	0.40	<0.001	<0.001
				Post	3	3	31	49	15			
		>30	73	Pre	19	26	38	16	0	0.93	<0.001	
				Post	3	8	38	42	8			

					Response*							
					**5**	**4**	**3**	**2**	**1**			

F	Smoke free public bars will make (have made) visits to them more comfortable	≤30	192	Pre	1	9	14	29	47	0.14	0.171	0.145
				Post	2	6	10	30	53			
		>30	73	Pre	3	11	19	33	34	0.36	0.044	
				Post	0	7	15	29	49			
G	The smoking ban will encourage (has encouraged) smokers to quit	≤30	192	Pre	1	7	19	54	20	-0.34	<0.001	0.087
				Post	4	10	27	49	10			
		>30	73	Pre	1	11	30	44	14	-0.12	0.472	
				Post	1	15	26	52	5			
H	The smoking ban is (was) needed to protect the health of workers	≤30	192	Pre	0	7	9	41	43	0.00	0.964	0.257
				Post	2	4	10	43	41			
		>30	73	Pre	1	14	22	37	26	0.12	0.358	
				Post	3	14	11	41	32			
I	Do you agree with the proposed ban on smoking in public bars?	≤30	192	Pre	5	9	13	27	47	0.15	0.313	<0.001
				Post	4	4	10	34	48			
		>30	71	Pre	24	23	1	28	24	0.76	0.002	
				Post	8	13	10	27	42			

*Response: 1-Strongly Agree, 2-Agree, 3-Undecided, 4-Disagree, 5-Strongly Disagree

** Wilcoxon-Mann-Whitney U-test of equal medians. Tests the change in attitude for each group.

*** Wilcoxon-Mann-Whitney U-test of equal medians. Tests the differences in change in attitude between groups.

### Smoking and attitude towards legislation

Before the legislation was introduced the non-smokers were generally more positive towards it than the smokers (Table 5). Patterns of change were very similar to those in Tables 2, 3 and 4. Questions A, C, D, E, F all showed significant or almost significant shifts in both smokers and non-smokers, while for questions B and I the shift was seen much more in the smokers, who had been initially less positive. Again, the only sizeable negative shift was on question G [see Additional file 1].

**Table 5:** Attitudes pre- and post-legislation split by smoking status group: for each question, percentages giving each response. N is the number of people in each smoking group.

	**Question**	**Smoking Status**	**N**	**Period**	Response*					**Mean Shift**	U-1** p-value	U-2*** p-value
					**1**	**2**	**3**	**4**	**5**			
A	The ban on smoking will have (has had) a negative effect on business for public bars	Smoker	108	Pre	24	35	27	13	1	1.06	<0.001	0.595
				Post	6	16	27	39	13			
		Non-Smoker	76	Pre	7	33	37	22	1	0.99	<0.001	
				Post	3	11	16	49	22			
B	The smoking ban is an unfair restriction on smokers	Smoker	107	Pre	21	23	16	32	7	0.29	0.093	0.088
				Post	10	26	19	34	11			
		Non-Smoker	76	Pre	5	3	17	33	42	0.00	0.967	
				Post	5	1	20	32	42			
C	Fewer people will (now) visit public bars after (because of) the ban on smoking	Smoker	108	Pre	19	33	24	21	2	1.08	<0.001	0.006
				Post	3	10	27	44	17			
		Non-Smoker	76	Pre	4	24	30	33	9	0.53	<0.001	
				Post	4	12	11	55	18			
D	The smoking ban will make (has made) smokers smoke more at home	Smoker	108	Pre	14	32	28	24	2	0.25	0.063	0.526
				Post	12	19	34	32	2			
		Non-Smoker	76	Pre	13	26	42	13	5	0.34	0.014	
				Post	5	13	55	24	3			
E	The smoking ban will result (has resulted) in jobs being lost	Smoker	108	Pre	14	15	31	37	3	0.63	<0.001	0.267
				Post	3	6	28	51	12			
		Non-Smoker	76	Pre	3	22	28	41	7	0.47	0.001	
				Post	4	3	25	53	16			

					Response*							
					**5**	**4**	**3**	**2**	**1**			

F	Smoke free public bars will make (have made) visits to them more comfortable	Smoker	108	Pre	4	17	24	37	19	0.35	0.017	0.35
				Post	1	11	19	39	30			
		Non-Smoker	76	Pre	0	3	11	20	67	0.21	0.049	
				Post	1	0	4	14	81			
G	The smoking ban will encourage (has encouraged) smokers to quit	Smoker	108	Pre	1	11	23	47	18	-0.22	0.079	0.54
				Post	2	15	26	49	8			
		Non-Smoker	76	Pre	1	5	25	58	11	-0.17	0.168	
				Post	1	11	29	51	8			
H	The smoking ban is (was) needed to protect the health of workers	Smoker	108	Pre	1	18	19	44	18	0.13	0.271	0.06
				Post	2	15	13	49	21			
		Non-Smoker	76	Pre	0	3	7	26	64	-0.09	0.713	
				Post	3	1	8	26	62			
I	Do you agree with the proposed ban on smoking in public bars?	Smoker	108	Pre	18	20	17	21	24	0.56	0.005	0.001
				Post	8	12	15	31	33			
		Non-Smoker	75	Pre	3	4	7	23	64	0.15	0.28	
				Post	3	1	4	21	71			

*Response: 1-Strongly Agree, 2-Agree, 3-Undecided, 4-Disagree, 5-Strongly Disagree.

**Wilcoxon-Mann-Whitney U-test of equal medians. Tests the change in attitude for each group.

***Wilcoxon-Mann-Whitney U-test of equal medians. Tests the differences in change in attitude between groups.

### Summary

Overall, bar workers were generally quite positive towards the smoking restrictions pre-implementation and, regardless of age and smoking status, they became even more positive at follow-up. Age and smoking status affected attitude pre-implementation, with the young and the non-smokers being more positive. The changes in attitude were seen in all smoking groups; there were differences by age group, with the initially more sceptical older group becoming rather less so after the implementation.

## Discussion

### Main results

The detrimental effects of smoking have been known for decades [23], and according to the Scottish Health Education Population Survey the general population are aware of the health risks associated with smoking, but may view smoking as a personal choice. Therefore the main thrust of current health education efforts is on raising awareness of the health risks of smoking to others and reducing the acceptability of second-hand smoke exposure [24]. To this end Scotland has followed the public health policies in many developed countries, in prohibiting smoking in enclosed public places in March 2006. Early indications are that smoke-free legislation in Scotland has greatly improved the air quality in bars, providing greater protection to bar workers and patrons from the harmful effects of second-hand smoke [25]. This study shows that bar staff were positive about the likely effects of the legislation before it was implemented, and became even more so afterwards.

The high level of agreement that smoke-free legislation was needed to protect bar workers' health may be due to the comprehensive information campaign (NHS Health Scotland, The Scottish Executive & Cancer Research UK) in the months leading up to the legislation. The generally positive experience of the legislation in Ireland may also have influenced Scottish bar workers' expectations and attitudes towards smoking restrictions [26].

One of the criticisms of the legislation has been the possibility that smoking would be displaced to the home. This concern was raised by the then Secretary of State for Health for England and Wales in September 2004 and is also reflected in the initial responses from the bar workers [27]. Their concern decreased by the time of the post-implementation interviews, when only 26% believed that displacement had occurred. However, an evaluation of the behavioural impact of the Irish smoke-free legislation found that the proportion of Irish homes with smoking bans increased [16].

Bar workers in this study expressed concern that the legislation might lead to economic losses for the pub trade, perhaps reflecting concerns expressed by the hospitality industry [28] rather than the experience of other countries, which have not seen trade affected adversely [29-31]. We note that there was a more positive attitude to business and job security at the follow-up survey.

The only topic where we observed a negative shift in attitudes towards the legislation was in relation to whether the legislation would encourage smokers to quit. This may reflect some bar workers' observations that they, their colleagues and patrons have continued to smoke. However, studies that have assessed changes in smoking habits since the introduction of smoke-free legislation report that the measures helped smokers quit [16].

While both smokers and non-smokers appeared to support the legislation, initially the attitudes of older bar workers were less positive than those of younger bar workers. The older workers generally demonstrated a greater change, perhaps reflecting a marked shift in social norms about the acceptability of smoking.

Those lost to follow-up were on average younger by three years. This reflects the fact that younger bar workers are more mobile and likely to have shorter periods of employment within the hospitality sector. The possibility for response bias was thus greater among the younger group, but it is clear from comparison of Tables 2 and 3 that the distribution of initial attitudes among those followed up was almost identical to that of the initial sample. We have no reason to believe that the reasons for loss to follow-up are linked with attitudes to smoking, or that the sample followed up are not representative of young bar workers generally. There was less non-response among the older group, representative of those with longer service and experience who are likely to benefit most from the legislation because of their reduced exposure to SHS over time, and who have the greatest role to play in its enforcement. Figures from Local Authorities (municipalities) show that, in the first few months following the Scottish legislation, a compliance rate in excess of 99 per cent was achieved [32]. The high compliance rates are testament to bar workers' positive attitudes towards the legislation as they performed the role of 'gate-keepers' in enforcing the legislation within their premises.

### Limitations and Further Research

The refusals rate of 55% of the bar managers approached at baseline is similar to that observed in other studies in this setting, [5,33] perhaps reflecting a protest against the impending ban. In recognition of the low take up rate at baseline our sampling strategy aimed to include a diverse sample of bars covering a broad range of socio-economic areas in urban, semi-urban and rural settings to provide an overall picture of the experience of legislation.

Further work to examine the attitudes of this group of bar workers is planned as part of the one-year follow-up of the BHETSE study. Some of our group are also involved in a similar study of bar workers as part of the evaluation of smoke-free legislation in England and it will be of interest to compare and contrast these two groups. Changes in respiratory health among the BHETSE cohort, and how these influence individual attitudes, may also be worth examining.

## Conclusion

### What this paper adds

This is the first evaluation of changes in bar workers' attitudes towards legislation prohibiting smoking in enclosed public places in Scotland. This study shows that bar staff were positive about the likely effects of the legislation before it was implemented, and became even more so afterwards. This positive change in attitudes may reflect a marked shift in social norms about the acceptability of smoking.

The high level of support for smoke-free legislation in Scotland is evidence of a continuing gradual shift in public attitudes towards the harmful effects of SHS exposure, and increasing acceptance that smoke-free environments are necessary to protect bar workers' and non-smokers' health. Changing social norms relate to community-wide behaviour, as distinct from the more direct influence of family and friends, and it seems possible that the high level of support for the smoke-free legislation may have arisen from a combination of positive media reporting and an intensive educational campaign from NHS Health Scotland, CRUK and the Scottish Executive in the months leading up to the legislation. This study demonstrates that smoke-free legislation is welcomed by staff as a public health measure, with young bar workers the most positive. The evaluation of future smoke-free legislation in other countries should seek to understand the concerns and attitudes of those stakeholders involved in implementation and enforcement. In Scotland bar workers played a central role in ensuring a high level of compliance and helped establish smoke-free entertainment venues as the new social norm.

## Competing interests

The author(s) declare that they have no competing interests.

## Authors' contributions

SS, JA participated in the design of their respective studies discussed in the workshop. SH, SS drafted the manuscript. BM & LM conducted the statistical analysis. BM, LM, MP & JA all commented on drafts of the manuscript. SH, SD & AN collected the data and all authors approved the final manuscript.

## Pre-publication history

The pre-publication history for this paper can be accessed here:



## Supplementary Material

Additional file 1 Data showing attitudes by age and smoking status. Tables of attitudes by age and smoking status. Click here for file
